
JOURNAL INFORMATION
==============================
NLM Title Abbreviation: Alcohol Res Health
Journal ID: Alcohol Res Health
NLM Journal ID: 100900708
Journal ID: ARH

Alcohol Research & Health

ISSN: 1535-7414
EISSN: 1930-0573
Publisher: National Institute on Alcohol Abuse and Alcoholism

ARTICLE INFORMATION
==============================
PMCID: PMC3860480
Article ID: arh-31-3-279
Article version: 1
Subjects: Glossary

Glossary

Print publication date: 2008
Volume: 31
Issue: 3
First page: 279
Last page: 283
Copyright year: 2008
License: Unless otherwise noted in the text, all material appearing in this journal is in the public domain and may be reproduced without permission. Citation of the source is appreciated.
License URL: https://creativecommons.org/publicdomain/mark/1.0/

Comment in: Strategies To Study The Neuroscience of Alcoholism: Introduction 31 3 2008 231 232 Alcohol Research & Health PMC3860475 23584864
Comment in: Communication Networks in the Brain 31 3 2008 196 214 Alcohol Research & Health PMC3860493 23584863
Comment in: Neurobiology of Alcohol Dependence 31 3 2008 185 195 Alcohol Research & Health PMC2770186 19881886

==============================
(18F)-fluoro-2-deoxyglucose (FDG): A positron-emitting analog of the sugar glucose; frequently used in positron emission tomography (PET) to measure brain activity.

95 percent confidence interval (CI): A statistical variable that can be used to determine how reliable the value of a parameter is that cannot be measured exactly based on a few measurements. The 95 percent CI gives an estimated range for the parameter that includes the actual value with a likelihood of 95 percent. The larger that range, the less reliable the estimated value.

γ-Aminobutyric acid (GABA): A neurotransmitter in the brain that acts to reduce the activity of the signal-receiving nerve cell (i.e., an inhibitory neurotransmitter); helps to restore homeostasis after a stressful situation.

Agonist: An agent that mimics the actions or effects of another agent (e.g., a drug that mimics the effects of a neurotransmitter).

Alcohol deprivation effect: Animal model of relapse in which animals are first chronically exposed to alcohol, followed by a period during which alcohol is withheld; if alcohol is then reintroduced, the animals consume more alcohol than they did before the enforced abstinence period.

Allele: One of two or more variants of a gene or other DNA sequences. Different alleles of a gene generally serve the same function (e.g., code for a protein that affects eye color) but may produce different phenotypes (e.g., blue eyes or brown eyes). Some alleles may be defective and produce a protein that has no function or an abnormal function.

Amino acid: Building blocks of proteins, characterized by the presence of an amino group (NH2), a carboxy group (COOH), and a side chain that differs for each amino acid; proteins are defined by their sequence of amino acids.

Amplitude: For a wave (or a variable that changes in a wave-like pattern), the “size” of the wave—that is, the difference between the lowest point (i.e., the trough) and the highest point (i.e., the peak).

Amygdala: Almond-shaped groups of nerve cells (i.e., neurons) located deep in the brain that are involved in the processing and memory of emotional reactions.

Analyte: Substance or chemical constituent that is determined in an analytical procedure.

Anisotropic: Not having properties that are all in the same direction; showing differences in a property or effect in different directions.

Antagonist: An agent that blocks or reverses the actions or effects of another agent (e.g., a drug that blocks the effects of a neurotransmitter).

Anterior: Toward the front (e.g., the anterior region of the brain is located toward the front of the head).

centiMorgan (cM): Standard unit of genetic distance on a genetic map, a measure of distance on the chromosomes; does not constitute a fixed number of nucleotides but is determined by how frequently two loci on the same chromosome are separated by recombination.

Cerebral spinal fluid: Fluid that surrounds the brain and spinal cord.

Cholinergic: Related to the neurotransmitter acetylcholine.

Chromatography: The collective term for a family of laboratory techniques to separate compounds in a mixture based on their differential movement through a two-phase system (e.g., a solid material and a liquid buffer solution).

Congenic animals (strains): Animal strains generated in the laboratory by mating two inbred strains, and backcrossing the descendants for 5–10 generations with one of the original strains (i.e., the recipient strain). After repeated back-crosses, the congenic strains carry the DNA of the recipient strain except for one relatively small piece of DNA that is still derived from the second parental inbred strain (i.e., the donor strain). Different congenic strains obtained from the same parental strains differ in which part of the donor DNA they still carry.

Corticotrophin-releasing factor (CRF): A hormone produced in the brain in response to stress that helps mediate the stress response.

Covalent modification: A chemical group (e.g., a phosphate or methyl group) that is attached to a protein via a covalent bond (i.e., a chemical bond between two molecules that involves sharing of electrons by atoms from both molecules) during or after translation.

Diffusion: Process by which molecules dissolved in a solution move from areas of higher concentration to areas of lower concentration.

DNA (deoxyribonucleic acid): The molecule that carries the genetic code in all organisms except some viruses. DNA is composed of a sequence of nucleotides.

Dopamine: Neurotransmitter that is involved, among other functions, in controlling behavior and cognition, motor activity, motivation and reward, mood, attention, and learning. Dopamine-producing nerve cells are located primarily in the ventral tegmental area (VTA) and in regions of the hypothalamus.

Dopaminergic: Referring to nerve cells that use dopamine as a neurotransmitter.

Dynamic range: A term used to describe the ratio between the smallest and largest possible values of a changeable quantity; for example, a test that can accurately measure the value of a variable within one order of magnitude has a smaller dynamic range than a test that can measure the value within three orders of magnitude.

Electroencephalogram (EEG): A test that measures and records the electrical activity of the brain; these waves are classified into different “bands” based on their frequency. Thus, δ waves have a frequency of up to 3 Hz, θ waves a frequency of 4 to 7 Hz, α waves a frequency of 8 to 12 Hz, β waves a frequency of 12 to 30 Hz, and gamma waves a frequency of 26 Hz and above. Different waves represent different brain functions.

Electrospray: An ionization strategy used to create a gas phase; charged analytes that can be separated based on their mass-to-charge ratio (m/z) and detected by mass spectrometry.

Endogenous opioids: A group of small molecules, such as endorphins and enkephalins, that are naturally produced in the body and which have effects similar to those of the opiates (e.g., morphine and heroin); endogenous opioids modulate the actions of various neurotransmitters.

Endophenotype: A heritable trait or characteristic that is thought to be an intermediate phenotype between a genetic predisposition and a clinical disorder; for example, certain neurobiological characteristics have been noted in people with alcoholism and may be used as endophenotypes to identify people at risk for alcoholism. Endophenotypes are thought to be useful for gene identification under the assumption that they are simpler and closer to the genetic underpinnings of the disorder.

Event-related potential (ERP): A characteristic pattern of brain waves elicited when a person is exposed to a sudden stimulus (e.g., a sudden sound or light).

Excitatory neurotransmitter: Any neurotransmitter that enhances the activity of the signal-receiving neuron.

Expression quantitative trait locus (eQTL): A quantitative trait locus (QTL) that does not contain a candidate gene thought to underlie the quantitative trait but a DNA element that controls the expression of such a candidate gene.

Fractionation: Separation process in which a mixture of compounds (e.g., proteins) is divided into smaller subsets (i.e., fractions) based on differences in a specific property of the compounds (e.g., solubility in water).

GABAergic: Transmitting or secreting the neurotransmitter γ-aminobutyric acid (GABA).

Gene expression: The process by which the genetic information encoded in the DNA is converted into a protein product.

Genome: The total genetic material of an organism, species, or cell.

Genotype: The complete genetic makeup of an organism determined by the particular combination of alleles for all genes; can also be used to refer to the combination of alleles for a particular gene.

Glutamate: A neurotransmitter that increases the activity of the signal-receiving nerve cell (i.e., an excitatory neurotransmitter).

Glutamatergic: Transmitting or secreting the neurotransmitter glutamate.

G-protein: Any of a family of intracellular regulatory molecules whose actions are triggered by the binding of certain neurotransmitters to their receptors. Several types of G proteins exist, including stimulatory G proteins that enhance the activities of other enzymes and inhibitory G proteins that inhibit the activities of other enzymes.

Gray matter: A generic term for a collection of neuronal cell bodies in the central nervous system.

Haplotype: A set of closely linked genetic markers present on one chromosome that tend to be inherited together.

Hippocampus: A part of the forebrain, located in the medial temporal lobe. It belongs to the limbic system and plays major roles in short-term memory and spatial navigation. Humans and other mammals have two hippocampi, one in each side of the brain.

Hydrophobicity: The physical property of a molecule that is repelled from water and does not dissolve in water.

Hypothermia: A condition in which an organism’s temperature drops below that required for normal metabolism and bodily functions.

In silico: Refers to an analysis or experiment performed using a computer or using a computer simulation.

In vivo: (Latin: within the living) Experimentation that takes place in or on the living organism.

Inbred strains: Animal strains generated by repeatedly inbreeding brother-sister pairs so that ultimately all animals of the strain are genetically identical.

Inhibitory neurotransmitter: Any neurotransmitter that in the brain acts to reduce the activity of the signal-receiving neuron.

Interval-specific congenic (ISC): Animals generated by crossing animals from two inbred strains and then repeatedly backcrossing offspring carrying a desired DNA segment into one of the parental strains. ISC animals have an identical genetic background and differ only in a relatively small segment of DNA (i.e., the congenic interval); in a panel of related ISC animals, the differing DNA segments overlap partially and together span a larger DNA region of interest.

ISC line (ISCL): A group of interval-specific congenic (ISC) animals with identical genetic background that all carry the desired small DNA piece (i.e., congenic interval) on one copy of the respective chromosome; the DNA on the other copy of the chromosome is inherited from the background strain into which the animals have been backcrossed.

ISC strain (ISCS): A group of interval-specific congenic (ISC) animals with identical genetic background that all carry the desired small DNA piece (i.e., congenic interval) on both copies of the respective chromosome.

Isotope: Any of the several different forms of a chemical element that differ in their atomic mass (mass number); isotopes of an element possess the same number of protons in their nuclei but different numbers of neutrons and therefore differ in their mass numbers, which reflect the total number of protons plus neutrons.

Lentivirus: A genus of slow-growing viruses, part of the Retroviridae family that possess RNA rather than DNA as their genetic material.

Lipoprotein: A compound molecule that contains both a protein and a lipid component.

Locus (pl. loci): The position a gene occupies on a chromosome.

Magnetic field gradient coil: A device to create a magnetic field whose strength increases or decreases in a linear pattern across different locations.

Mass selection: The selective stabilization of an ion of a specific mass-to-charge ratio (m/z) in a magnetic or electric field.

Mass spectrometry (MS): An analytical technique that identifies the chemical composition of a compound or sample based on the mass-to-charge ratio of charged particles. For MS, isolated proteins are broken down into peptides and the masses of these peptides are determined through one of several approaches. Based on the measured masses of all peptides, computers can determine the most likely protein from which they were derived.

Mesolimbic dopamine system: System of interconnected brain regions consisting of the ventral tegmental area, nucleus accumbens, and components of the limbic system, such as the amygdala; is considered the brain’s reward pathway that mediates the rewarding effects associated with alcohol and other drug use as well as other experiences and is therefore a central factor in the development of addiction.

Messenger RNA (mRNA): Key intermediary molecule generated during gene expression; mRNA levels for a gene are used as an indicator of how “active” the gene is (i.e., how much of the protein is produced).

Meta-analysis: An analysis that combines the results of several studies addressing a set of related research hypotheses.

Microarray: A chip made from glass, plastic, or other type of support material onto which a large number of minute samples (e.g., proteins, DNA samples) are affixed in an orderly manner for performing automated assays.

Microsatellites: Highly polymorphic loci found every few thousand nucleotides in the DNA that can be used to determine from which parent or ancestor a specific DNA sequence has been inherited. Microsatellites typically consist of short sequences of 1 to 6 nucleotides that can be repeated 10 to 100 times. Each person or animal has a specific pattern of microsatellites that can be used to determine inheritance patterns.

Multidimensional separation method: As applied to chromatography, a separation approach that uses more than one physical property (e.g., hydrophobicity and charge) for biochemical separation; conversely, single-dimensional separation methods use only one physical property for separation (e.g., hydrophobicity).

Neuroactive steroid: Any of a group of steroid molecules that are naturally produced in the adrenal glands, ovaries, and testes and which can act on neurons and modulate their functions.

Neuroplasticity: Ability of the nervous system to change and reorganize itself throughout life by forming new connections among nerve cells or altering the activities of existing nerve cells and connections; is the basis of the ability to learn throughout life.

Neurotransmitter: Signaling molecules produced in nerve cells (i.e., neurons) that serve to transmit signals from one neuron to another neuron or from a neuron to another type of cell (e.g., muscle cell); neurotransmitters are released from the signal-emitting neuron and bind to receptors on the surface of the signal-receiving cell.

Nucleotide: A subunit of DNA and RNA consisting of a nitrogen-containing base, a phosphate group, and a sugar molecule.

Nucleus accumbens (NAc): A structure in the brain that is composed mainly of nerve cell bodies and which acts as a transit point for nerve signals in specific neural subsystems; located in the forebrain, plays an important role in reward, pleasure, addiction, and fear; primarily contains GABAergic neurons.

Oscillation: The repetitive variation, typically in time, of a parameter about a central value (e.g., by a pendulum).

Parietal lobe: Part of the brain that is located at the top and to the sides of the brain, behind the frontal lobe; integrates sensory information from different organs, determines spatial sense and navigation.

Peak abundance: The signal response of an ionized molecule that is detected by an analytical instrument.

Peak capacity: The maximum number of analytes that can be resolved by a separation strategy (e.g., mass spectrometry or chromatography). The greater the resolution of the mass spectrometer or the chromatographic separation, the more analytes can be distinguished from one another and the higher the peak capacity.

Peptide: A small molecule composed of a short chain of the building blocks of proteins (i.e., amino acids); in contrast, proteins are defined as long chains of amino acids.

Pharmacodynamics: The study of the biochemical and physiological effects of drugs on the body, the mechanisms of drug action, and the relationship between drug concentration and effect.

Pharmacokinetics: The study of how substances that are administered from the outside are processed in the body (i.e., taken up [absorbed], metabolized, and excreted).

Phenotype: The observable structural or functional characteristics of an individual organism that result from the interaction of its genotype with environmental factors.

Pleiotropic effect: Multiple effects from a single gene.

Polymorphism: The presence of two or more alleles of a gene or other DNA sequence in a population.

Positron: Positively charged particle that has the same mass as an electron; may be generated by a type of radioactive decay; release of these particles is measured during positron emission tomography (PET).

Positron emission tomography (PET): Imaging technique that produces a three-dimensional image based on signals (i.e., positrons) emitted by a radioactive tracer molecule that is introduced into the body in a variant of a biologically active molecule (e.g., (18F)-fluoro-2-deoxyglucose [FDG]).

Posterior: Toward the back (e.g., the posterior region of the brain is located toward the back of the head).

Proteomics: The large-scale study of the structure and function of proteins.

Quantitative trait: A phenotypic characteristic that varies in the degree to which it is present (e.g., sensitivity to alcohol or height) and which typically is determined by more than one gene as well as environmental factors..

Quantitative trait locus (QTL): A DNA region that is associated with a quantitative trait and which may contain one of the genes contributing to that trait.

Receptor: A protein molecule located in the membrane surrounding a cell or in the cell’s interior that can bind with a signaling molecule (e.g., a hormone or neurotransmitter), thereby setting off a chain of biochemical reactions in the cell that alter the cell’s behavior in a specific manner.

Recombinant inbred (RI) mouse strains: Sets of animal strains derived from the same two parental inbred strains that each carry a specific combination of the parental genes; within each RI strain all animals are genetically identical.

Recombination: Rearranement of genetic material during the production of the germ cell that results in a unique combination of genes in each individual, appears more commonly to occur at certain sites on the DNA (hot spots) that would be expected by chance alone.

RNA (ribonucleic acid): Macromolecule related in structure to DNA from which it differs in the type of sugar found in each nucleotide; typically a single-stranded molecule. Different types of RNA molecules are found in human cells and cells of other organisms (e.g., messenger RNA [mRNA]). Some viruses (e.g., lentivirus) use RNA rather than DNA as their genetic material.

Selected lines: Lines of animals generated by selectively breeding those animals in a population that have either very high or very low levels of a phenotype of interest (e.g., voluntary alcohol consumption); after several generations of selective breeding, the resulting lines will exhibit stable differences in the phenotype of interest; in contrast to inbred strains that also may differ in a certain phenotype, the animals within a selected line are not genetically identical.

Selected reaction monitoring (SRM): A mode of tandem mass spectrometry where a selected precursor ion and product ion are monitored. This approach is highly selective and only molecular species with the desired combination of the precursor and product ion mass-to-charge ratio (m/z) are transmitted to the detector.

Shotgun proteomics: Strategy for protein analysis using mass spectrometry at the peptide level. Proteins in a sample are first digested into peptides and then analyzed by mass spectrometry. A list of the original proteins in the sample then is deduced by reassembling the peptides back into proteins in silico.

Short-interfering RNA (siRNA): Artificially generated short RNA molecules that in contrast to normal RNA molecules are double-stranded; the nucleotide sequence of the siRNA is designed so that it can interact with a specific messenger RNA (mRNA), and thus causing the degradation of the mRNA, and by silencing the corresponding gene.

Single-nucleotide polymorphism (SNP): A variation in the DNA sequence of the genome that occurs when a single DNA building block (i.e., nucleotide) differs between members of a species.

Small-hairpin RNA (shRNA): Artificially generated short RNA molecules whose nucleotide sequence is designed so that it forms a double-stranded arm with a hairpin turn at one end; can be used to silence gene expression by interfering with the messenger RNA (mRNA).

Stable isotopes: Nonradioactive elements whose nuclei contain the same number of protons but different numbers of neutrons. Molecules made with stable isotopes are commonly used as quantitative internal standards because chemically they are almost identical to the corresponding natural molecules.

Striatum: A part of the brain that is involved in the planning of movement as well as in other cognitive processes; in humans the striatum is activated by stimuli associated with reward, but also with aversive, novel, unexpected, or particularly intense stimuli; includes several nuclei, including the nucleus accumbens.

Tandem mass spectrometry (MS/MS): Analytic technique to identify proteins; involves multiple steps of mass spectrometry (MS) selection, with some form of fragmentation occurring in between the steps so that increasingly smaller protein fragments are analyzed.

Tandem mass spectrometer: A mass spectrometer that can perform more than one stage of mass selection. The stages of mass selection can be performed in space (e.g., using a triple quadrupole mass spectrometer) or in time (e.g., using an ion trap mass spectrometer).

Tandem mass spectrum (MS/MS spectrum): A mass spectrum that is produced by using two steps of mass selection. The mass-to-charge ratio (m/z) of a peptide precursor ion is selected and fragmented into product ions. The m/z of the product ions then are separated, detected, and displayed.

Transcription: Biochemical process in which messenger RNA (mRNA) is generated based on the genetic information of the DNA.

Transcription factor: A protein that regulates gene expression by binding to specific sites on the DNA near the start of the gene, thereby activating other proteins involved in transcription.

Transcriptome: The entirety of all transcription products (transcripts or messenger RNA [mRNA]) present in a cell, tissue, or organism.

Transcriptomics: The large-scale study of the expression level of messenger RNA (mRNA) in a given cell, organ, or organism.

Transfection: The introduction of foreign material into eukaryotic cells using a virus vector or other means of transfer.

Translation: Biochemical process during which messenger RNA (mRNA) serves as a blueprint based on which proteins are synthesized from their building blocks, amino acids.

Triple quadrupole mass spectrometer: A mass spectrometer with a linear series of three quadrupole mass analyzers to separate ions. The first (Q1) and third (Q3) quadrupoles act as mass filters, and the middle (Q2) quadrupole is used as a collision cell for analyte fragmentation.

Vector: A vehicle (e.g., a virus or a short piece of DNA called a plasmid) used to transfer foreign genetic material into a cell.

Ventral tegmental area: Area located in the midbrain that contains dopaminergic nerve cell bodies which project to various parts of the forebrain, including the nucleus accumbens and other parts of the mesolimbic dopamine system.

Visual oddball paradigm: In a typical oddball experimental design, subjects must perform a task (e.g., press a button) when they see a rare target stimulus that appears 20 percent of the time (e.g., a red box) interspersed among a series of common nontarget stimuli that appear 80 percent of the time (e.g., a green box).

Voxel: A volume element, representing a value on a regular grid in three-dimensional space.

White matter: A generic term for a collection of nerve cell fibers (i.e., axons) in the central nervous system.

